# Improved motion correction for T_1 _mapping

**DOI:** 10.1186/1532-429X-16-S1-P45

**Published:** 2014-01-16

**Authors:** Sébastien Roujol, Murilo Foppa, Keigo Kawaji, Kraig V Kissinger, Beth Goddu, Warren J Manning, Reza Nezafat

**Affiliations:** 1 Medicine, BIDMC/Harvard Medical School, Boston, Massachusetts, USA; 2 Radiology, BIDMC/Harvard Medical School, Boston, Massachusetts, USA

## Background

Quantitative myocardial T_1 _mapping is commonly performed using a breath-hold ECG-triggered acquisition. Despite breath-hold instructions, motion is observed in ~50% of patients due to diaphragmatic drift and their limited breath-holding capability [1]. Registration of each T_1_-weighted (T_1_w) image can be performed to reduce motion artifacts in T_1 _maps but remains challenging due to the high intensity variations among T_1_w images [1]. In this study we propose a novel non-rigid T_1_w image registration approach.

## Methods

Our proposed method uses an extended formulation of the optical flow problem, where both motion field and intensity variation are estimated simultaneously within a unified variational framework [2]. An additional term was introduced to constrain the deformation field using automatic feature point tracking [3]. Each T_1_w image is registered to the 4^th ^image of the series (reference), on which a region of interest is manually drawn around the left ventricle (LV-ROI). All remaining steps are performed automatically, where affine motion parameters are first estimated by maximization of the mutual information between the reference image and each T_1_w image over the LV-ROI, and is followed by our proposed non rigid motion estimation step. Twenty patients (57 ± 14 y, 12 m) referred for clinical CMR exams were scanned before and after administration of contrast agent. T_1 _mapping was performed in 1-3 slices with a 5-(3)-3 scheme for pre-contrast and 4-(1)-3-(1)-2 scheme for two post-contrast scans at ~15 and ~30 min post-injection. 85 total T_1 _maps were acquired and were visually assessed for the presence of motion. To quantify the registration step, the myocardium was manually segmented in all T_1_w images and the DICE coefficients were computed between each registered T_1_w image and the reference image (1: ideal match, 0: none). Overall T_1 _map quality and motion artifacts were assessed by a blinded reader using a 4-point scale (0: non diagnostic/severe motion artifact, 4: excellent/no motion artifact).

## Results

**Figure 1:**
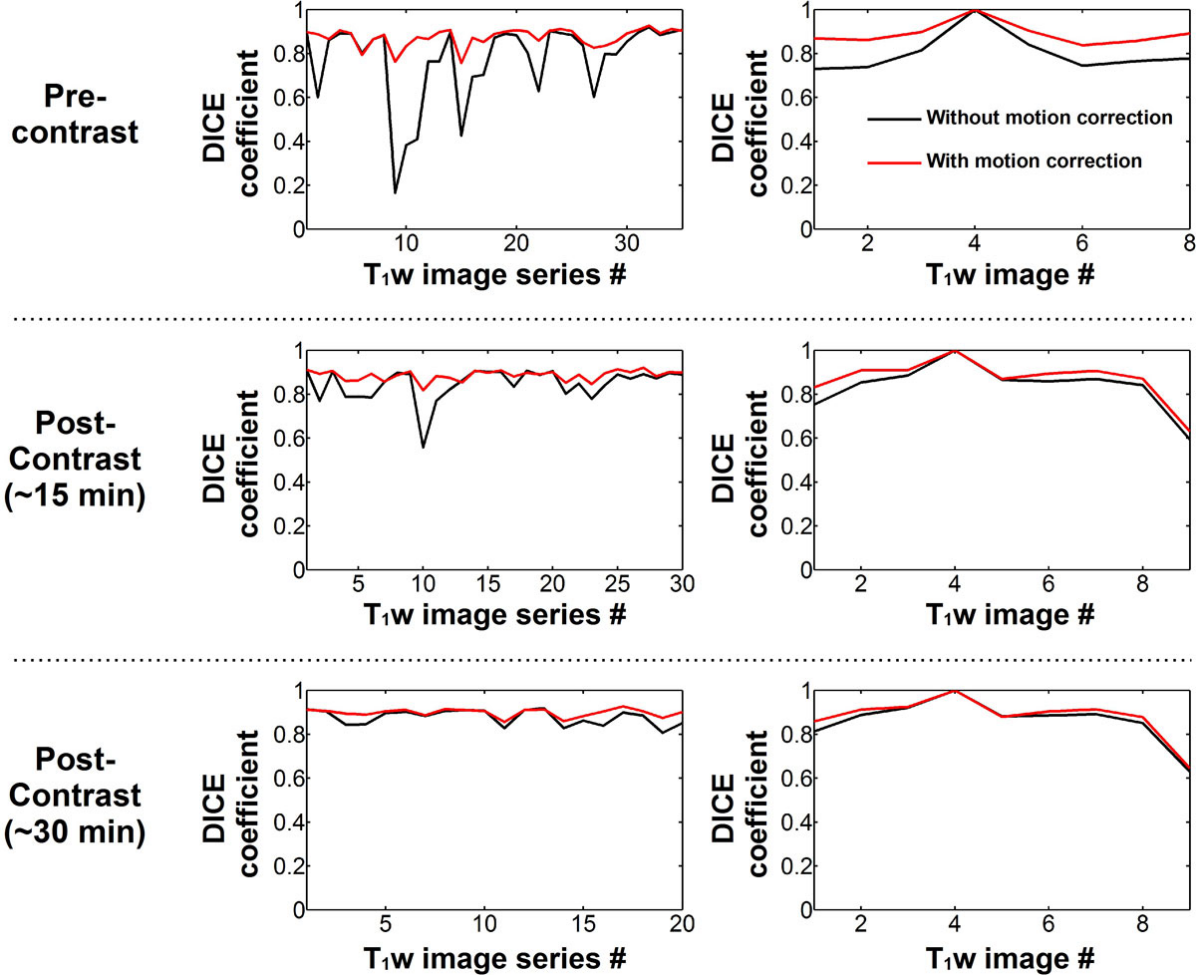
**DICE similarity coefficients obtained with and without the proposed motion correction approach in pre (5-3 scheme) and post contrast (4-3-2 scheme) T_1_w image series**. Average DICE coefficients over each T_1_w image series (left column), and over each individual T_1_-weighted image (right column) are shown. Similar DICE coefficients were observed before and after motion correction in T_1_w image series with high pre-registration coefficients (~0.9). Substantial improvements in DICE coefficient values were achieved in T_1_w image series with low pre-registration coefficients (< 0.9).

**Figure 2:**
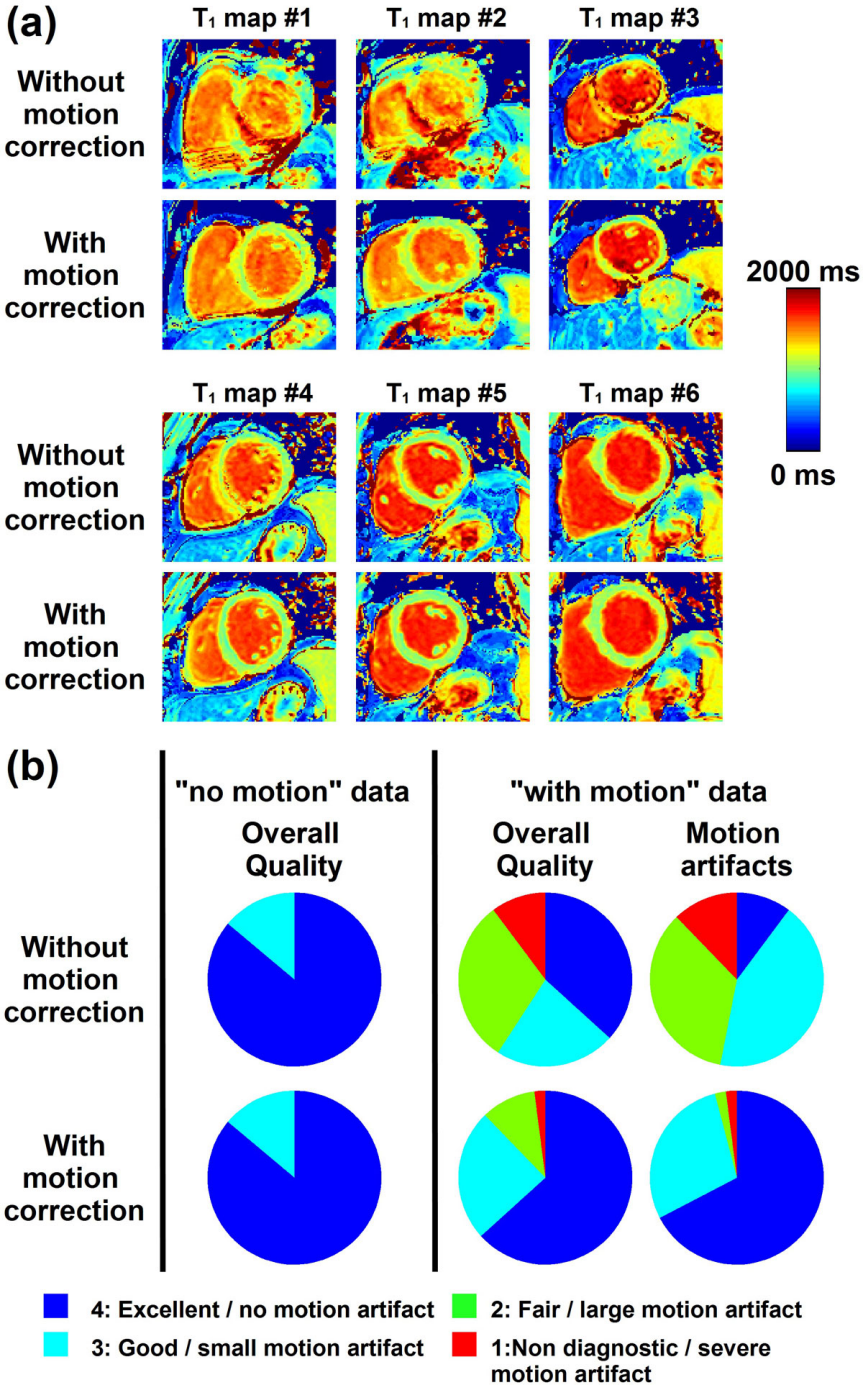
**Analysis of T_1 _maps before and after motion correction**. Examples of 6 T_1 _maps reconstructed before and after motion correction are shown in (a). Moderate to severe motion artifacts are visible in all shown T_1 _maps reconstructed without motion correction. After motion correction, the quality of all T_1 _maps significantly improved and motion artifacts were substantially reduced. Subjective quantitative analysis are shown in (b). Similar T_1 _map quality is observed before and after motion correction in data identified as "no motion". Improved image quality and reduced motion artifacts were observed after motion correction in data identified as "with motion".

57% of the T_1_w image series were visually identified as "with motion". After motion correction, DICE coefficients (Figure 1) were slightly improved in "no motion" series (0.90 ± 0.02 vs. 0.91 ± 0.02, p < 0.002) and greatly improved in "with motion" series (0.80 ± 0.14 vs. 0.89 ± 0.03, p < 0.001). Figure 2 shows T_1 _maps reconstructed with and without motion correction. No statistical difference was found in term of overall T_1 _map quality before and after correction in "no motion" series After motion correction, improved overall T_1 _map quality (2.86 ± 1.04 to 3.49 ± 0.77, p < 0.001) and reduced motion artifacts (2.51 ± 0.84 to 3.61 ± 0.64, p < 0.001) were obtained in "with motion" series.

## Conclusions

The proposed non-rigid registration approach reduces the respiratory-induced motion occurring during breath-hold T_1 _mapping and significantly improves the quality of T_1 _maps.

## Funding

NIH R01EB008743-01A2.

## References

[B1] Xue MRM 2012

[B2] Cornelius ACM-Siggraph 1983

[B3] Roujol IEEE-TITB 2012

